# Responsive‐Hydrogel Aquabots

**DOI:** 10.1002/advs.202401215

**Published:** 2024-07-29

**Authors:** Shipei Zhu, Huanqing Cui, Yi Pan, Derek Popple, Ganhua Xie, Zachary Fink, Jiale Han, Alex Zettl, Ho Cheung Shum, Thomas P Russell

**Affiliations:** ^1^ Materials Sciences Division Lawrence Berkeley National Laboratory One Cyclotron Road Berkeley CA 94720 USA; ^2^ Department of Mechanical Engineering The University of Hong Kong Pokfulam Road Hong Kong P. R. China; ^3^ Institute of Biomedical Engineering College of Medicine Southwest Jiaotong University Chengdu 610031 P. R. China; ^4^ Department of Physics University of California Berkeley Berkeley CA 94720 USA; ^5^ Department of Chemistry University of California Berkeley Berkeley CA 94720 USA; ^6^ State Key Laboratory for Chemo/Bio‐Sensing and Chemometrics College of Chemistry and Chemical Engineering Hunan University Changsha 410082 P. R. China; ^7^ Department of Polymer Science and Engineering University of Massachusetts Amherst Amherst MA 01003 USA; ^8^ Department of Materials Science and Engineering University of California Berkeley Berkeley CA 94720 USA; ^9^ Advanced Biomedical Instrumentation Centre Hong Kong Science Park Shatin, New Territories Hong Kong (SAR) 999077 P. R. China; ^10^ Advanced Institute for Materials Research (AIMR) Tohoku University 2‐1‐1 Katahira, Aoba Sendai 980‐8577 Japan

**Keywords:** all‐liquid robots, aqueous two‐phase systems, adaptive materials, flexible electronics, responsive hydrogels

## Abstract

It remains a challenge to produce soft robots that can mimic the responsive adaptability of living organisms. Rather than fabricating soft robots from bulk hydrogels,hydrogels are integrated into the interfacial assembly of aqueous two‐phase systems to generate ultra‐soft and elastic all‐aqueous aquabots that exhibit responsive adaptability, that can shrink on demand and have electrically conductive functions. The adaptive functions of the aquabots provide a new platform to develop minimally invasive surgical devices, targeted drug delivery systems, and flexible electronic sensors and actuators.

## Graphical abstract

All‐water‐based aquabots functionalized with responsive hydrogel membranes are ultrasoft and elastic. They can adaptively shrink their size to navigate through much narrower spaces. Also, their electrically conductive membranes enable the fabrication of flexible electronic sensors and actuators.

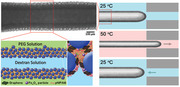

## 1 Introduction

Marine organisms, in response to internal or external stimuli, can change shape to adapt to a complex and ever‐changing environment.^[^ ^1^ ^]^ Octopi, for example, can squeeze into very narrow spaces to mate, feed on prey, or escape from predators.^[^ ^2^ ^]^ Blowfish, on the other hand, explosively inflate to ward off predators. Such responsive adaptability requires the integration of sensors and actuators without compromising the mechanical flexibility necessary to realize rapid deformation. To mimic the adaptive functions of living organisms, much effort has been focused on producing sensory‐responsive soft actuators or robots. Adding sensing functionality to current elastomeric soft robots has required physically connecting individual, often bulky components by multi‐step integration processes, such as welding,^[^ ^3^ ^]^ 3D printing,^[^ ^4^ ^]^ embedding,^[^ ^5^ ^]^ or laminating.^[^ ^6^ ^]^ These physically integrated components not only give rise to stress concentration and adhesion issues,^[^ ^7^ ^]^ but also restrict motility and miniaturization.^[^ ^8^ ^]^ It is highly desirable, therefore, to integrate multi‐functionality in soft robots at the molecular rather than the system level, mitigating the need for bulky components.^[^ ^7^, ^9^, ^10^ ^]^ Cross–linked, water‐swollen polymer gel networks can be made to respond to changes in temperature, light,^[^ ^11^ ^]^ and pH^[^ ^12^ ^]^ by volumetric or solubility changes brought about by configurational changes of the polymer chains in the network. For example, water‐soluble coiled structures of poly(*N*‐isopropylacrylamide) (PNIPAM) can transform to water‐insoluble globular structures when heated to temperatures above the lower critical solution temperature (LCST).^[^ ^13^, ^14^ ^]^ This contraction causes an expulsion of water from the PNIPAM hydrogel and, therefore, a volume contraction or shrinkage occurs. Being aqueous‐based, these responsive hydrogels are attractive for water‐based chemical and biological reactions, as well as the encapsulation and release of biological molecules.^[^ ^15^, ^16^ ^]^ Recently, multi‐material responsive hydrogels have been patterned into soft magnetic actuators from lipid droplet networks.^[^ ^7^ ^]^ Pre‐gel lipid droplets are photopolymerized to generate thermally responsive PNIPAM hydrogels. The lipid bilayers on the droplet interface function as volume‐defining enclosures during the polymerization, leading to stable, continuous hydrogels. Different responsive additives, such as NIPAM monomers, gold nanoparticles (AuNPs), and magnetic particles, can be added to the system prior to gelation and can then be patterned into multi‐material hydrogels that are responsive to a range of external stimuli, including temperature, light, and magnetic fields.^[^ ^7^ ^]^ Thermally and magnetically responsive hydrogel grippers, for example, that can shrink and curl for cargo transport through channels narrower than the unshrunk hydrogels, have already been made.^[^ ^7^ ^]^ Responsive hydrogels are also used for millimeter‐scale soft robots capable of multimodal locomotion, photothermal responsiveness, and optical camouflage.^[^ ^17^ ^]^ The photothermal‐responsive function of soft robots can be realized by embedding gold nanorods (AuNRs) into bulk hydrogel films where photothermally triggered shrinkage enables the soft robot to pass through channels much smaller than their original size.^[^ ^17^ ^]^ More recently, a soft somatosensitive actuator capable of intrinsic piezoresistive sensing and photo/thermal actuation was developed based on an electrically responsive hydrogel,^[^ ^8^ ^]^ composed of an interpenetrating polymer double‐network of PNIPAM and polyaniline (PANI). The PNIPAM network afforded thermo‐responsive actuation and mechanical flexibility, while the conductive PANI acts as both a photothermal transducer and a piezoresistive sensor.^[^ ^8^ ^]^ Here we describe the integration of stimuli‐responsive hydrogels into aquabots,^[^ ^18^ ^]^ nanoparticle‐polyelectrolyte stabilized all‐aqueous robots recently developed in our laboratories, to generate adaptive, multiple stimuli‐responsive soft robots incorporating a shrink‐on‐demand function.

Responsive hydrogel‐based soft actuators or robots are fully solidified bulk structures that lack micron‐scale compartmentalization, necessary for cascading reaction systems typically found in natural systems. In addition, the deformability of these soft robots is limited by the underlying bulk rheology, and the extent of shrinkage is restricted by the water content. Rather than using a bulk fabrication method of the hydrogels, we combined aqueous phase‐separation‐induced photopolymerization and all‐aqueous 3D printing to overcome these challenges. The hierarchical compartmentalized tubular and multicellular structures of printed aquabots are functionalized by a responsive hydrogel membrane during the aqueous phase separation. Two‐photon polymerization (2PP) employs a tightly focused femtosecond laser to photo‐polymerize resin precursors to produce arbitrary and ultraprecise 3D microstructures with high resolution.^[^ ^19^ ^]^ 2PP is rapidly developing to fabricate high‐resolution, microstructured soft actuators.^[^ ^20^ ^]^ However, the resolution relies on the precision of movement of the focused beam in the photoresist according to a computer‐designed 3D route. In our system, we harness the aqueous phase separation to generate the internal hierarchical microstructures of aquabots rather than manipulating the movement of light beam. The functionalized aquabots respond to a range of stimuli and pass through spaces much smaller than their original sizes by an on‐demand, reversible shrinkage that can be further enhanced by the higher water content in the compartmentalized structures.

## 2 Results and Discussion

**Figure 1:**
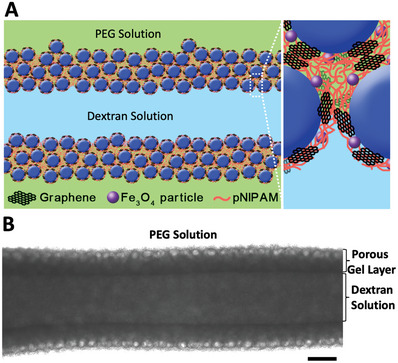
Building the thermal responsive membrane of aquabots. A) Schematic of hierarchical structures of a printed aquabot. B) Printed aquabot with hierarchical structures. The scale bar is 100 µm.

The ability to direct components osmotically suggests a new way to functionalize the aquabots. If the osmotically controlled components, such as thermal responsive molecules (*N*‐isopropylacrylamide (NIPAM) monomers), can be selectively located at different positions in the droplet during osmotic transport, the elapsed time after osmosis begins should determine the final morphology of the solidified structures. To test this hypothesis, a droplet of an aqueous solution having 10 wt.% dextran (Mw = 10 000 g mol^−1^) and 5 wt.% polyethylene glycol (Mw = 8000 g mol^−1^) dissolved in water containing graphene nanosheets, *N,N'*‐Methylenebisacrylamide (MBAm) cross‐linking agents, NIPAM monomers, and photo‐initiator molecules is placed in a continuous phase of an aqueous solution of 25 wt.% polyethylene glycol (Mw = 8000 g mol^−1^) dissolved in water. The droplet osmolality (160 mOsm kg^−1^) is lower than that of the continuous phase (1050 mOsm kg^−1^). UV illumination triggers the polymerization of NIPAM into PNIPAM and solidifies the droplet. Various solidified structures can be observed, depending on the waiting time prior to UV illumination, as shown in Figure S1 (Supporting Information). When the droplets are exposed to UV immediately after droplet formation, all monomers stay within the droplets and a totally solidified hydrogel particle is formed. If UV exposure is delayed 5 s, the majority of monomers have been driven to the interface of the droplet, and a capsule is formed with a wall thickness that decreases with the time allowed for osmotic transport. When the droplets are illuminated after a delay time of 120 s, the droplet cannot be solidified since the monomers have diffused out of the droplet, and only graphene nanoplatelet aggregates are observed at the interface. Graphene nanoplatelets driven by the capillary force tend to segregate on the interface, leading to a Janus droplet partially covered by nanoplatelets. The osmotic flow of water is further visualized by magnetic particles driven to the droplet interface, as shown in Figure S2 (Supporting Information). This demonstrates that osmosis is key in defining the structures of the synthetic membranes. These structures are akin to what we reported previously.^[^ ^21^ ^]^ If the droplet solution is jetted into the continuous phase, non‐equilibrated shapes can be produced by UV‐assisted all‐aqueous 3D printing. The entire system is photocured after the completion of printing. To print a homogenous morphology, the printing speed is set as rapid as 5 cm /s and the printing time of a linear tubule is <1 s, which can be negligible in comparison to the delay time of illumination. If the UV illumination is delayed 5 s after printing, the photopolymerizable molecules will flow to the interface and a hollow tubular structure is formed. As shown in **Figure** **1**, an aquabot with a porous membrane functionalized with thermally responsive PNIPAM and magnetic particles can be produced and even the phase‐separated sub‐droplets on the interface are stabilized by the photo‐cross–linking. The thickness of the membrane decreases if the delay time is 10 s instead of 5 s. If the delay time is >15 s, the liquid thread will not be stabilized. To adjust the time window for photocuring that can maintain the designed morphology, we can tune the concentration of NIPAM monomers and the intensity of UV light. To print more complicated structures that take longer times, we can design photomasks to precisely control the delay time of illumination for different parts of the aquabots.

**Figure 2:**
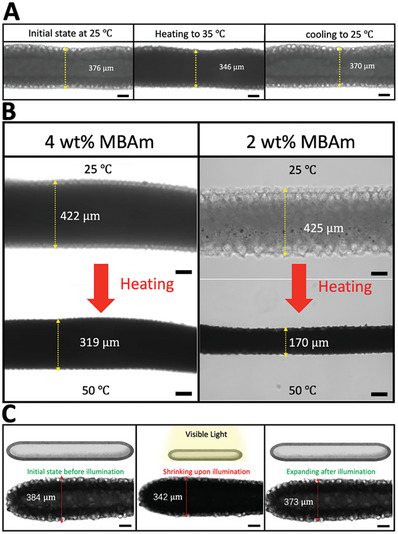
On‐demand shrink ability of aquabots. A) Aquabot shrinks upon heating and expands to its original size upon cooling. B,C) Enhancing the shrinkage of aquabots by decreasing the degree of photo‐cross–linking. C) Reversible photothermal shrinkage of AuNR‐ or GNP‐modified aquabots triggered by illumination of visible light at room temperature. All scale bars are 100 µm.

To test the reversibility of the shrinking of the aquabots made from PNIPAM, the diameter of the aquabot is measured upon heating and cooling. As shown in **Figure** **2**A, upon heating, the diameter shrinks from 376 to 346 µm at 35 °C, which is above the LCST of PNIPAM 32 °C. Upon cooling, the diameter then expands to close to its original size at 25 °C, which is below the LCST. Both the size and hierarchical compartmental structures, including the sub‐capsules in the membrane, of the aquabot are preserved during the reversible shrinking. The extent of shrinkage of the aquabots determines the minimum dimensions of the channel through which the aquabot can pass. The more water that is squeezed from the aquabot, the smaller it will be. To enhance the shrinkage, the water content in the photo‐cross–linked network can be increased by decreasing the degree of photo‐cross–linking. As reported previously,^[^ ^22^ ^]^ PNIPAM hydrogels undergo two stages of shrinking. Initially, water molecules are more mobile in the free volume of polymer matrices, the water content decreases more obviously. In the later shrinking process, it is more difficult to extract the water molecules absorbed into the polymer chains, and the water content decreases much more slowly. In our system, the looser polymer network is produced by decreasing the degree of photo‐cross–linking, and thus the more mobile water molecules in the free volume of the porous hydrogel membrane can be easily squeezed out during the shrinking process. As shown in Figure 2B, upon the heating from 25 to 50 °C, the diameter of an aquabot made from a 4 wt.% concentration of photo‐cross–linking agents shrinks from 422 to 319 µm (24.4%‐dimensional reduction); while the diameter of an aquabot made from a 2 wt.% concentration of photo‐cross–linking agents shrinks from 425 to 170 µm (60%‐dimensional reduction). The dimensional shrinkage ratio of multiple aquabots fabricated from different concentrations of cross–linkers (2 and 4 wt.% MBAm) is shown in Figure S3A (Supporting Information). The average dimensional shrinkage ratio for 2 wt.% MBAm is 53.8% with a standard deviation of 4.8%, while the average dimensional shrinkage ratio for 4 wt.% MBAm is 25.9% with a standard deviation of 4.9% (Figure S3B, Supporting Information). The dimensional shrinkage ratio achieved is larger than the 45% reported for highly shrinkable phase‐separated PNIPAM‐hydrogels.^[^ ^23^ ^]^ The extent of dimensional shrinkage is dramatically enhanced by decreasing the degree of photo‐cross–linking. In practical applications, aquabots should be shrink‐on‐demand and not dependent on the temperature of the external environment. Without changing the external temperature, a photothermal strategy provides a means of varying the local temperature within the aquabot, triggering a shrinkage on demand. To do this, gold nanorods (AuNRs) and graphene nanoplatelets (GNPs) are used as photothermal materials and are dispersed in the ink phase before printing. The membrane of the aquabot is then functionalized with AuNRs and GNPs after the phase‐separation‐induced self‐assembly. Once the robot is exposed to the high intensity of visible light, AuNRs will absorb the light by a localized plasmon surface resonance (LPSR) at specific wavelengths, imparting photoacoustic and photothermal properties.^[^ ^17^, ^24^ ^]^ Due to the photothermal effect, the local temperature of the irradiated region will increase above the LCST of PNIPAM hydrogels, inducing the shrinkage of aquabots in water with a surrounding temperature of ≈25 °C. Upon exposure to the light source, the diameter shrinks from 384 to 342 µm (≈11% shrinkage); while upon the removal of the light source, the robot recovers its original size (Figure 2C; Video S1, Supporting Information).

**Figure 3:**
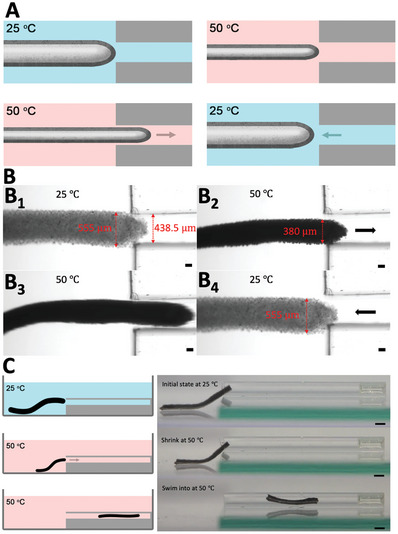
Shrink‐on‐demand aquabot passing through narrow spaces. A) Schematic and B) experimental scenarios of an aquabot navigating through the 2D channel narrower than its original size. Scale bars are 100 µm. C) Aquabot navigating through the 3D confined narrow space upon shrinking. Scale bars are 1 mm.

The aquabots can navigate through a space narrower than their original size by harnessing this shrink‐on‐demand characteristic. An aquabot with an initial 555 µm diameter cannot enter a narrower 438.5 µm wide channel, as shown in **Figure** **3**A,B1. To minimize the contact friction between the aquabot and the wall of the channel, the diameter of the aquabot was then reduced to 380 µm upon heating (Figure 3B2; Video S2, Supporting Information), and the aquabot was actuated in one direction by using a gradient magnetic field when its size was much smaller than that of the channel. it can smoothly enter the channel (Figure 3B3). After exiting the channel, the robot then swells to its original size upon cooling (Figure 3B4). After entering, the entire body of the shrink‐on‐demand aquabot can pass through the narrower channel, as shown in Figure 3C. This ability enables the aquabot to adjust its size in situ to accomplish tasks, such as targeted drug delivery and endoscopic surgery, in the narrower space. The minimization of aquabots can also be finely controlled by the printing conditions. The diameter of printed liquids stabilized by interfacial assembly can be easily adjusted by the flow rate of the ink, diameter of the printing nozzle, and the printing speed,^[^ ^25^ ^]^ as shown in Figure S4 (Supporting Information). Aquabots with diameters as small as 100 µm were achieved.^[^ ^18^ ^]^

**Figure 4:**
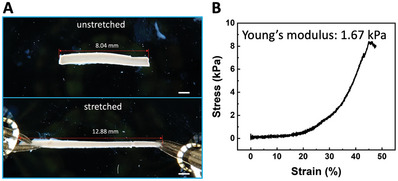
A) Stretched PNIPAM hydrogel‐membrane‐based all‐water robot under the force loading of two tweezers. The stretching ratio $\lambda =\frac{12.88}{8.04}=1.6.$ B) Measured stress‐strain curve of PNIPAM hydrogel‐membrane‐based all‐water robot. Scale bars are 1 mm.

To verify whether the elasticity of PNIPAM hydrogel‐membrane‐based aquabots is enhanced, the mechanical properties were measured with an Instron equipped with sensitive force sensors (Figure S5, Supporting Information). Based on the measured stress‐strain curve (**Figure** **4**B), the yield stress can be as high as 9 kPa, much higher than that of polyelectrolyte‐nanoparticle membrane‐based aquabots (540 Pa).^[^ ^18^ ^]^ The maximum stretching ratio is as high as λ  =  *L*/ *L*_0_ =  1.45, where *L* is the length of stretched aquabot, *L*_0_ is the original length. The Young's modulus is only 1.67 kPa, indicating the ultrasoftness of the aquabot. By manually stretching the aquabot using tweezers, we were able to achieve λ  =  1.6 (Figure 4A) by varying the concentration of cross–linkers. The structure of our aquabots contains a liquid channel and a porous hydrogel membrane. The liquid in the channel is fluidic and makes a negligible contribution to the elasticity of the aquabot. The elasticity of aquabots only relies on the hydrogel membrane. The Young's modulus of our hydrogel membrane (1.67 kPa) is much smaller than that of the bulk hydrogel (37.24 kPa),^[^ ^17^ ^]^ due to its phase‐separated porous structure and looser polymer network.

**Figure 5:**
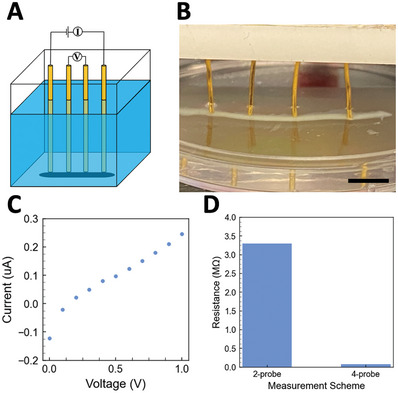
Resistance measurements for conductive PANI‐PNIPAM double‐network hydrogel‐membrane‐based aquabots. A) Schematic of 4‐probe resistance measurement. B) 4‐probe resistance measurement. The neighboring probe distance is 0.5 cm. C) *I–V* curve of 2‐probe resistance measurement. D) Comparison of resistances measured by 2‐probe and 4‐probe methods. Scale bar is 5 mm.

To build electronic sensors for somatosensitive aquabots, conductive polymers are used to functionalize the PNIPAM membrane. Water‐soluble sulfonated conductive polyaniline (PANI) is dissolved in the ink. Water soluble, conductive poly(3,4‐ethylenedioxythiophene):poly(styrene sulfonate) (PEDOT:PSS) is dissolved in the matrix phase. The PNIPAM network is first rapidly photopolymerized to form the membrane during printing. The PEDOT:PSS and sulfonated PANI molecules assemble into a conductive composite network at the water‐water interface, due to the electrostatic interactions between the ${\mathrm{SO}}_{3}^{-}$ groups of the PSS and the protonated NH^+^ groups of the PANI and the strong π − π stacking interactions between PEDOT and PANI.^[^ ^26^ ^]^ The conductive PEDOT:PSS/PANI composite network encapsulates the PNIPAM network. To remove the ions from the aqueous solution, the aquabots are washed with DI water and transferred to DI water for the measurement of electrical conductivity. Both 2‐point‐probe and 4‐point‐probe resistance measurements are used to verify the electrical conductivity of the membrane, as shown in **Figure** **5**A,B. The resistance normalized by length of the 2‐point‐probe measurement is (Figure 5C,D) as high as 3.3 MΩ cm^−1^. Deionized water can easily form an insulating liquid layer between the probes and the aquabot. Another insulating layer is from microbubbles of oxygen and hydrogen around the probes generated from the electrolysis of water. For a robust contact to reduce the high contact resistance, the probes can be immersed into the ink droplet prior to the printing, and conductive hydrogels will grow in situ around the probes to create direct contact areas. We can also increase the direct contact area by increasing the diameter of probes or aquabots. The 4‐point probe measurement effectively removes the contact resistance and provides the intrinsic measurement of the resistance of the membrane.^[^ ^27^ ^]^ The normalized resistance of the 4‐point‐probe measurement is only 78.4 kΩ cm^−1^ and the conductivity of the aquabot is calculated to be 0.65 S m^−1^ based on the size (length = 1 cm and diameter = 0.5 mm), which is comparable to the conductivity (2.46 S m^−1^) of the highly conductive ITUC hydrogels synthesized by an ice‐templated, UV polymerization of PNIPAM and cryo‐polymerization of PANI.^[^ ^8^ ^]^

## 3 Conclusion

In conclusion, diverse hierarchical structures of responsive hydrogels can be tuned by photopolymerizing osmotically‐driven components at different aqueous‐phase‐separated states. By harnessing the conditions for producing a responsive hydrogel‐membrane interface, hierarchical multi‐compartmental architectures of aquabots are produced. Adaptive, shrink‐on‐demand aquabots are produced by functionalizing their walls with complex hydrogel membranes that are responsive to temperature, light, and magnetic fields. Due to the higher water content of the all‐aqueous system, the dimensional change of the aquabots can be enhanced while maintaining their ultra‐softness without compromising the elasticity, enabling the robot to adapt its size and shape in situ to navigate through and conduct tasks in spaces, much narrower than the size of the robot. With the assistance of medical imaging tools, such as x‐ray imaging and ultrasound imaging that are used to track the movement of magnetic soft robots inside the biological body in precise control,^[^ ^28^, ^29^, ^30^ ^]^ ultrasoft and elastic hydrogel aquabots can adapt their size by photothermal shrinking to pass through tortuous and narrow vessels in tissues; thus the systems hold significant potential in applications for targeted drug delivery and microsurgery in vivo. To realize inhomogeneous responsiveness in the 3 directions, the liquid‐in‐liquid 3D printing technology will be used to print more complicated shapes with flexible geometries and gradient components of responsive materials. Also, the magnetization profile of our aquabots containing ferromagnetic particles can be easily programmed to achieve multimodal locomotion, as demonstrated in elastomeric soft robots.^[^ ^31^ ^]^ The conductive hydrogels can be easily assembled at the water‐water interface and made electrically conductive to generate electronic sensors for somatosensitive aquabots. Enhanced strain‐sensing properties of the conductive hydrogel contribute to both greater change in the relative resistance under stress and wider response to dynamic and static stimuli by adding poly(vinylidene fluoride‐*co*‐trifluoroethylene) (PVDF‐TrFE).^[^ ^32^ ^]^ To realize the piezoelectric sensing application, we can introduce PVDF‐TrFE to the conductive membrane of aquabots by a one‐pot cross‐linking method, as demonstrated in bulk composite hydrogels.^[^ ^32^ ^]^ The liquid‐in‐liquid 3D printing can be used to program flexible patterns of liquid circuits. Multi‐step chemical transformations can be conducted within the printed liquid channels underflow, and can selectively transport materials across the liquid−liquid interface for in‐line separations, as demonstrated previously.^[^ ^33^, ^34^ ^]^ The coupling of the ion‐selective function, the electrically conductive properties, and piezoelectric properties have the potential to build all‐fluidic devices that can execute complex tasks, such as physiochemical and piezoelectric sensing, in a logical manner.

## Conflict of Interest

H.C.S. is a scientific advisor of EN Technology Limited, MicroDiagnostics Limited, PharmaEase Tech Limited, and Upgrade Biopolymers Limited in which he owns some equity, and a managing director of the research center Advanced Biomedical Instrumentation Center Limited. The works in this paper are however not directly related to the works of these entities, as far as we know.

## Supporting information

Supporting Information

Supplemental Video 1

Supplemental Video 2

## Data Availability

The data that support the findings of this study are available from the corresponding author upon reasonable request.
