# Supplementary material for: Responsive‐Hydrogel Aquabots
Source: Adv Sci (Weinh). 2024 Jul 29;11(36):2401215. doi: 10.1002/advs.202401215 (PMC11422812; doi:10.1002/advs.202401215)
Supplement: Supplementary file 1 — Supporting Information [file ADVS-11-2401215-s001.pdf]

ADVANCED SCIENCE
Open Access

Supporting Information

for *Adv. Sci.*, DOI 10.1002/advs.202401215

Responsive-Hydrogel Aquabots

Shipei Zhu, Huanqing Cui, Yi Pan, Derek Popple, Ganhua Xie, Zachary Fink, Jiale Han, Alex Zettl, Ho Cheung Shum and Thomas P Russell**

Supporting Information
©Wiley-VCH 2021
69451 Weinheim, Germany

Responsive-Hydrogel Aquabots

S. Zhu, H. Cui, Y. Pan, D. Popple, G. Xie, Z. Fink, J. Han, A. Zettl, H.C. Shum*, T.P. Russell*

Abstract: It remains a challenge to produce soft robots that can mimic the responsive adaptability of living organisms. Rather than fabricating soft robots from bulk hydrogels, we integrate responsive hydrogels into the interfacial assembly of aqueous two-phase systems to generate ultra-soft and elastic all-aqueous aquabots that exhibit responsive adaptability, being able to shrink-on-demand and having electrically conductive functions. The adaptive functions of aquabots provide a new platform to develop minimally invasive surgical devices, targeted drug delivery systems, and flexible electronic sensors and actuators.

DOI: 10.1002/anie.2021XXXXX

Experimental Procedures

Materials: N,N'-Methylenebisacrylamide (MBAm) cross-linking agents, N-Isopropylacrylamide (NIPAM), and photo-initiators 2,2'-Diethoxyacetophenone (DEAP) are purchased from Sigma Aldrich. Deionised (DI) water is used in all experiments. Iron oxide (II, III) (Fe3O4) nanoparticles of 100 nm diameter embedding in the membrane are purchased from Aladdin. Graphene nanoplatelets (GNPs) are purchased from TANFENG graphene Tech Co., Ltd. (China). The solutes of the nonequilibrium all-aqueous systems consists of polyethylene glycol (PEG) (Mw=8000 g/mol, Aladdin, Shanghai, China) and Dextran (Mw=10000 g/mol, CASB, Shanghai, China). hydrochloric acid and cetyltrimethyl ammonium bromide (CTAB) are obtained from Sinopharm Chemical Reagent Co., Ltd. Chloroauric acid, silver nitrate and hydroquinone are purchased from Aladdin. Poly(3,4-ethylenedioxythiophene):poly(styrene sulfonate) (PEDOT:PSS) conductive screen printable ink with 5 wt% concentration are purchased from Sigma Aldrich.

Synthesis of AuNRs: A growth solution containing 0.1 M hydrochloric acid and CTAB, 0.4×10^{-3} M chloroauric acid, 0.21×10^{-3} M silver nitrate, and 5.26×10^{-3} M hydroquinone was maintained at 30 °C in an incubator. The longitudinal localized surface plasmon resonance (LSPR) peak position of AuNRs is dominated by their aspect ratio, which can be regulated by the silver ion concentration (*Chem. Mater.* 2014, 26, 5, 1794–1798). We would like to fine-tune the aspect ratio of AuNRs by adding silver ion with an optimized concentration. The growth of AuNRs was initiated by slowly adding 0.0017×10^{-3} M sodium borohydride and was allowed to continue for 8 h. The AuNRs were purified by three precipitation-centrifugation cycles (8500 rpm, 15 min) and redispersed in 1 mL of water.

Solutions of ink phase:

NIPAM monomers (809×10^{-3} mol/L), MBAm cross-linking agents (44.08×10^{-3} mol/L) and DEAP photo-initiators (3×10^{-6} mol/L) are pre-dissolved in aqueous ink phase made from 10 wt% Dextran and 5 wt% PEG before printing and UV polymerization.

Synthesis of sulfonated PANI solution:

Initially hydrochloric acid (37% Sigma–Aldrich, 14.78 mL) and aniline monomer (Sigma–Aldrich, 16.76 g) were dissolved in deionized water (600 mL) and the solution was stirred vigorously in an ice bath maintained at 5°C in a 1 L flask. After 30 minutes of stirring, the polymerization reaction was initiated with ammonium persulfate (Sigma–Aldrich, 0.18 mol in 60 mL deionized water), which was gradually added into the flask over 30 minutes. The reaction mixture was then stirred for 3 hours at 5°C. The resulting emeraldine salt precipitate was filtered and washed thoroughly with deionized water and methanol. To convert the emeraldine salt to the emeraldine base, ammonium hydroxide (Sigma–Aldrich, 1 M) was stirred into the filtered solution at room temperature. The product was filtered and dried under vacuum in an oven at 60 °C overnight, yielding a blue powder. Briefly, for every 0.5 g of dried emeraldine base, phenylhydrazine (Sigma–Aldrich, 2.5 mL) was added and mixed in a glass mortar. The mixture was pressed with a glass pestle for 10 minutes and subsequently stirred for one hour to complete the reducing reaction, before being diluted with ethyl ether (Sigma–Aldrich, 75 mL). The product (leucoemeraldine base) was filtered and washed with ethyl ether, then suction dried. The sulfonation reaction was carried out using precooled (5 °C) fuming sulfuric acid (Sigma–Aldrich, 10 mL), which was added to the dried leucoemeraldine base in the mortar and stirred for 1 hour in an ice bath. The reaction was terminated by slow addition of the sulfonated PANI (S-PANI) into ice water, which precipitated the S-PANI polymer. The S-PANI precipitates were cleaned and filtered using a vacuum funnel, after which the filter cake was dispersed at a high concentration in water. Subsequently, dialysis was performed for 1 week while changing water two times a day using a Snakeskin dialysis tubing MWCO 5,000 (Thermo Fischer Scientific) to remove impurities and unreacted aniline and initiator. Following dialysis the concentrated S-PANI solution was diluted to 10 mg/mL using distilled water and stored at room temperature.

Experimental Setups

Heating plate: The liquid bath containing aquabots is placed on a transparent plate with adjustable heating temperature.

Microscopy: The on-demand shrink behaviors of aquabots are monitored by a microscope (DMIL LED Fluo, Leica) equipped with a camera (Infinity 3, Lumenera) processed by ImageJ (NIH) software.

Osmolarities measurement: The osmolarities of the droplet phase and the continuous phase were measured by osmometer (Model 3320, Advanced Instrument, Inc.).

Visible light source: The optical fiber connection with the Xenon light source (400 mW, LS-3000UV, QSPEC, China) is applied to trigger the photothermal shrinking of all-water robots. The light intensity is 5500 lux. The wavelength of the white light source is 400-700 nm, which is not limited to a single wavelength.

3D Printing: Aquabots were printed by a commercially available QidiTech 3D printer, with the print heads replaced by a syringe connected to a air pump. The syringe was covered by aluminium foil to avoid the photopolymerization of ink source in the syringe.

UV light source for photopolymerization: During the 3D printing, the NIPAM is polymerized via irradiation with UV (wavelength 365 nm, irradiation Intensity 25 mW/cm^2) SPOT-CURE SP-VI (Ushio Inc.) equipment for a specific time to get the desired hollow structures of the aquabots containing PNIPAM layer. The dish containing the matrix phase of printing is immersed in the ice bath to remove the heat from the UV light source.

Mechanical properties measurement:
Tensile tester Instron (Zwick Roel) was used to record the tensile response of stretched aquabots. Variations in the resistance of aquabots during uniaxial tensile tests were recorded by a digital source meter (2450; Keithley Instruments).

***DC* conductivity measurements:** A Keithley 2400 SourceMeter was used for all DC measurements. The gold probes were cut to size from commercially available gold wires (99.999%). 2-probe measurements were conducted with an applied potential of 50 mV to avoid side reactions in the solvents. For 4-probe measurements, a plastic dish was used to contain the DI-water solutions. Two gold probes contacted the ends of printed aquabot were used to supply a current of ±100 μA, while other two gold probes located in the middle region of aquabot were used to measure the induced voltage. The conductivity was calculated according to the formula: $\sigma = L/(R\pi r^2)$, where R is the resistance, L is the length of aquabot and r is the radius of aquabot.

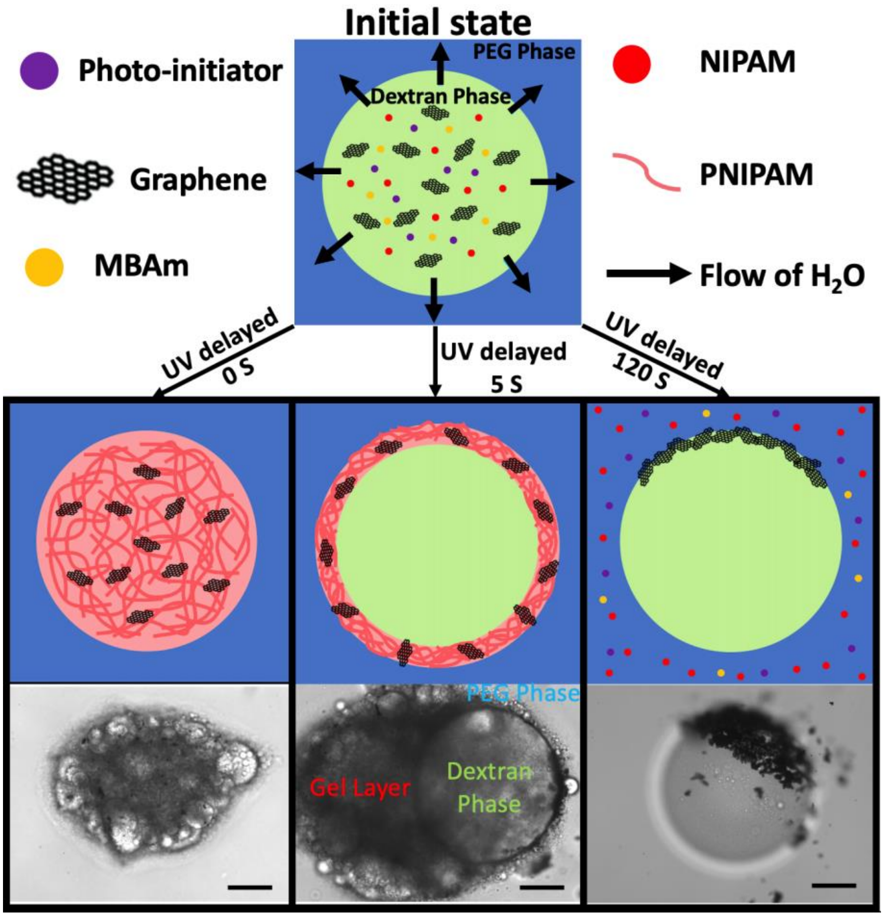

Figure S1. Diverse morphologies induced by the osmotic flow and UV solidification. Scale bars are 100 μm.

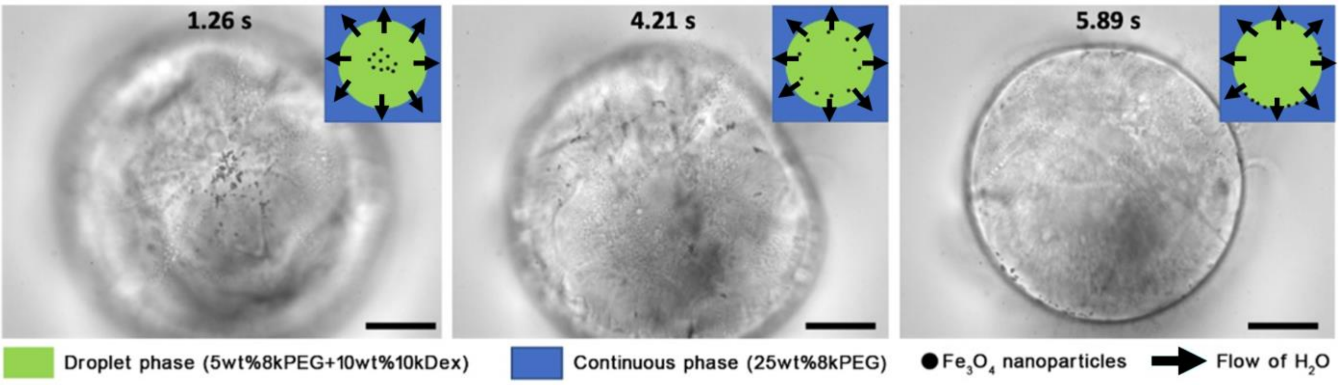

Figure S2. Time-lapse optical microscope images and corresponding schematics indicating the osmotically driven flow of particles to the interface. Scale bars are 100 μm.

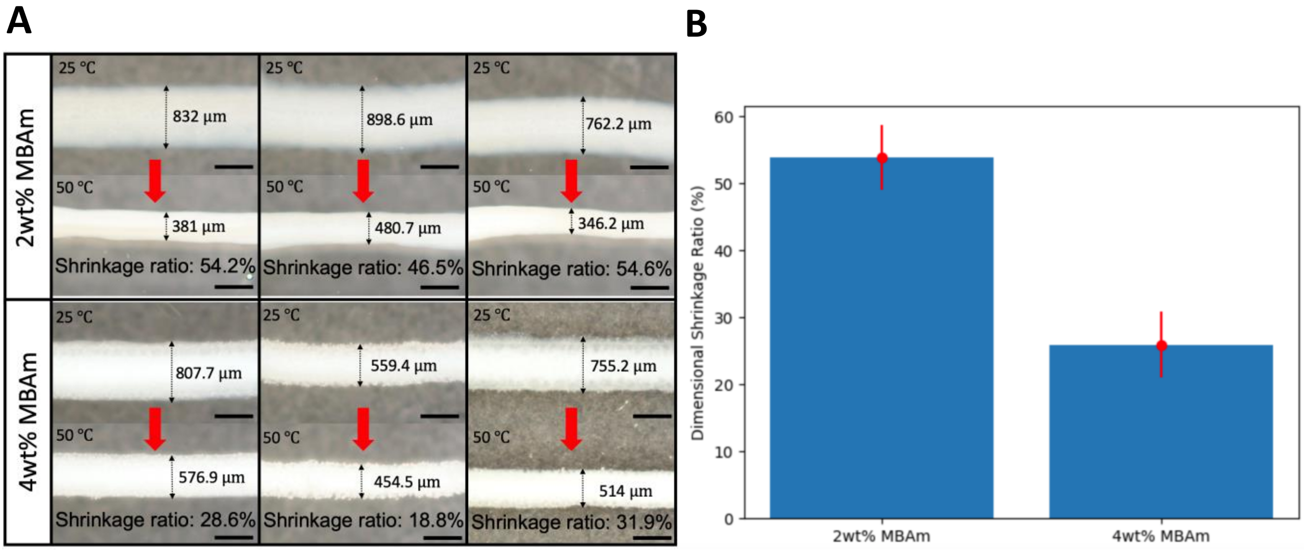

Figure S3. (A) Measurement of dimensional shrinkage ratio of multiple aquabots fabricated from 2wt% and 4wt% MBAm crosslinkers. (B) The average dimensional shrinkage ratio and standard deviation calculated from measured values. Scale bars are 500 μm.

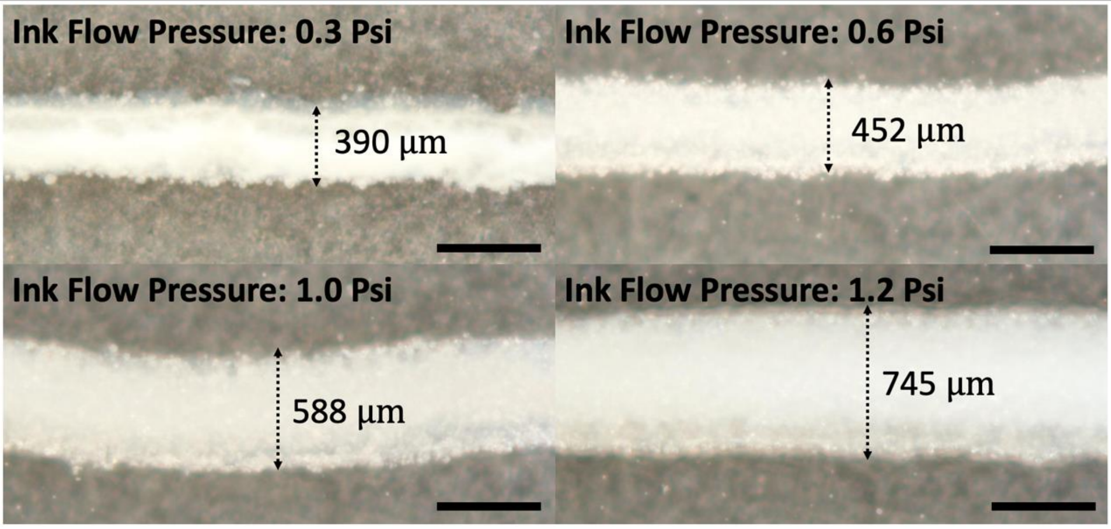

Figure S4. Diameter of printed aquabots tuned by the ink flow rate regulated by flow pressure of air pump. The diameter of printing nozzle is 500 μm and the speed of printing head is 10 mm/s. Scale bars are 500 μm.

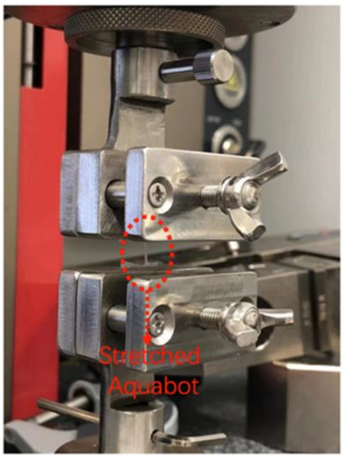

Figure S5. Stretched PNIPAM-hydrogel membrane-based all-water robot under the force loading of an Instron tensile tester.
